# Effect of GA_3_ Treatment on Seed Development and Seed-Related Gene Expression in Grape 

**DOI:** 10.1371/journal.pone.0080044

**Published:** 2013-11-05

**Authors:** Chenxia Cheng, Xiaozhao Xu, Stacy D. Singer, Jun Li, Hongjing Zhang, Min Gao, Li Wang, Junyang Song, Xiping Wang

**Affiliations:** 1 College of Horticulture, State Key Laboratory of Crop Stress Biology in Arid Areas, Northwest A&F University, Yangling, Shaanxi, China; 2 Key Laboratory of Horticultural Plant Biology and Germplasm Innovation in Northwest China, Ministry of Agriculture, Northwest A&F University, Yangling, Shaanxi, China; 3 Department of Agricultural, Food and Nutritional Science, University of Alberta, Edmonton, Alberta, Canada; Instituto de Biología Molecular y Celular de Plantas, Spain

## Abstract

**Background:**

The phytohormone gibberellic acid (GA_3_) is widely used in the table grape industry to induce seedlessness in seeded varieties. However, there is a paucity of information concerning the mechanisms by which GAs induce seedlessness in grapes.

**Methodology/Principal Findings:**

In an effort to systematically analyze the cause of this GA_3_-induced seed abortion, we conducted an in depth characterization of two seeded grape cultivars (‘Kyoho’ and ‘Red Globe’), along with a seedless cultivar (‘Thompson Seedless’), following treatment with GA_3_. In a similar fashion to the seedless control, which exhibited GA_3_-induced abortion of the seeds 9 days after full bloom (DAF), both ‘Kyoho’ and ‘Red Globe’ seeded varieties exhibited complete abortion of the seeds 15 DAF when treated with GA_3_. Morphological analyses indicated that while fertilization appeared to occur normally following GA_3_ treatment, as well as in the untreated seedless control cultivar, seed growth eventually ceased. In addition, we found that GA_3_ application had an effect on redox homeostasis, which could potentially cause cell damage and subsequent seed abortion. Furthermore, we carried out an analysis of antioxidant enzyme activities, as well as transcript levels from various genes believed to be involved in seed development, and found several differences between GA_3_-treated and untreated controls.

**Conclusion:**

Therefore, it seems that the mechanisms driving GA_3_-induced seedlessness are similar in both seeded and seedless cultivars, and that the observed abortion of seeds may result at least in part from a GA_3_-induced increase in cell damage caused by reactive oxygen species, a decrease in antioxidant enzymatic activities, and an alteration of the expression of genes related to seed development.

## Introduction

Grapevine (*Vitis vinifera* L.) is one of the most widely cultivated and economically important fruit crops in the world. Seedlessness of the fruit, which occurs when grapes only produce traces of aborted seeds, no seeds, or a significantly reduced number of seeds, is an especially desirable trait for consumers in the table grape industry [1]. This aberration in seed development is caused by parthenocarpy or stenospermocarpy [2], with incomplete seed development occurring as a result of embryo abortion and endosperm breakdown caused by changes in physiological conditions brought on by high levels of growth-promoting phytohormones near or at bloom [3,4]. 

One such class of endogenous phytohormone is the gibberellic acids (GAs), which are tetracyclic diterpenoids that are essential to plant growth and development. GAs are involved in many aspects of plant growth, including stem and leaf elongation, flower induction, trichome, anther, fruit and seed development, and seed germination [5-10]. In addition to their roles in plant development, GAs have also been shown to have an effect on reactive oxygen and antioxidant activities within grape tissues [11,12].

Interestingly, many studies have demonstrated that seedlessness in grapes can be induced through the exogenous application of GA_3_. For example, Delaware grapevine (*V. labrusca*) produces seedless berries following the application of 100 ppm GA_3_ solution 12-17 d before full bloom [13], with similar effects being noted in Muscat Bailey A (*V. vinifera × V. labrusca*) [14], as well as Emperatriz and Emperador (*V. vinifera*) cultivars [15]. This role of GAs is not restricted to grapes, but has been observed in many species. For example, application of GA has been found to induce seed abortion in sweet cherry and Clementine mandarin [16,17], while GAs (possibly GA_1_ and/or GA_3_) have also been shown to play an important role early in pea (*Pisum sativum* L.) and *Arabidopsis thaliana* seed development by regulating embryo and/or endosperm and pollen tube development [9,18,19]. 

Although GAs are used widely in the table grape industry in all major producing countries, there is a paucity of information concerning the mechanisms by which GAs induce seedlessness in grapes. While a handful of studies have been carried out on this subject, the majority of these have focused upon the effect of GAs on pollen tube growth. These studies have demonstrated that GA_3_ applied to grape flowers before or during anthesis severely inhibits pollen germination and pollen tube growth [14], and this may possibly be due to the biosynthesis of pollen tube inhibitor(s), leading to the production of unfertilized ovules [20]. 

In an attempt to improve our understanding of the mechanisms by which GAs induce seedlessness in grape, we aimed to investigate the effect of pre-bloom GA_3_ application on the size and shape of berries and seeds, the frequency of seedlessness in the berries, and embryonic development. In addition, we conducted an analysis of the transcript levels of genes related to seed development following treatment with GA_3_. Furthermore, since GAs have been shown to have an effect on reactive oxygen species (ROS), which at high levels signal programmed cell death [21], as well as antioxidant enzyme activities within grape tissues [11,12], we tested whether GA_3_-induced seed abortion was linked to changes in ROS, cell damage and/or antioxidant enzyme activities.

## Results

### GA_3_-induced seedlessness in grape

To produce seedless grape berries in seeded cultivars (‘Kyoho’ and ‘Red Globe’), floral clusters were treated with GA_3_ 18 d prior to full bloom in the case of ‘Kyoho’ and 16 d prior to full bloom in the case of ‘Red Globe’ (Figure S1). As shown in Table 1, there was a significant increase in the incidence of seedless berries in both cultivars following GA_3_ treatment compared to untreated controls. In the case of ‘Kyoho’, the frequency of seedless berries was 3.3% and 98.6% in control and GA_3_-treated plants, respectively, whereas ‘Red Globe’ generated 3.0% and 85.2% seedless berries in control and GA_3_-treated plants, respectively. 

**Table 1:** Seedless berry frequency in GA_3_-treated and untreated control ‘Kyoho’ and ‘Red globe’ cultivars.

Cultivar	Treatment	Seedless berry frequency
‘Kyoho’	GA_3_ 100mg/L	98.6 ± 0.8**
	Control	3.3 ± 1.0
‘Red Globe’	GA_3_ 100mg/L	85.2 ± 5.0 **
	Control	3.0 ±1.5

Values of seedless berry frequencies represent the means ±SE of three replicates.

** Mean percentages derived from treated and untreated plants of the same cultivar were significantly different (P < 0.01).

### Effect of GA_3_ treatment on the growth of berries and seeds at different developmental stages

**Figure 1:**
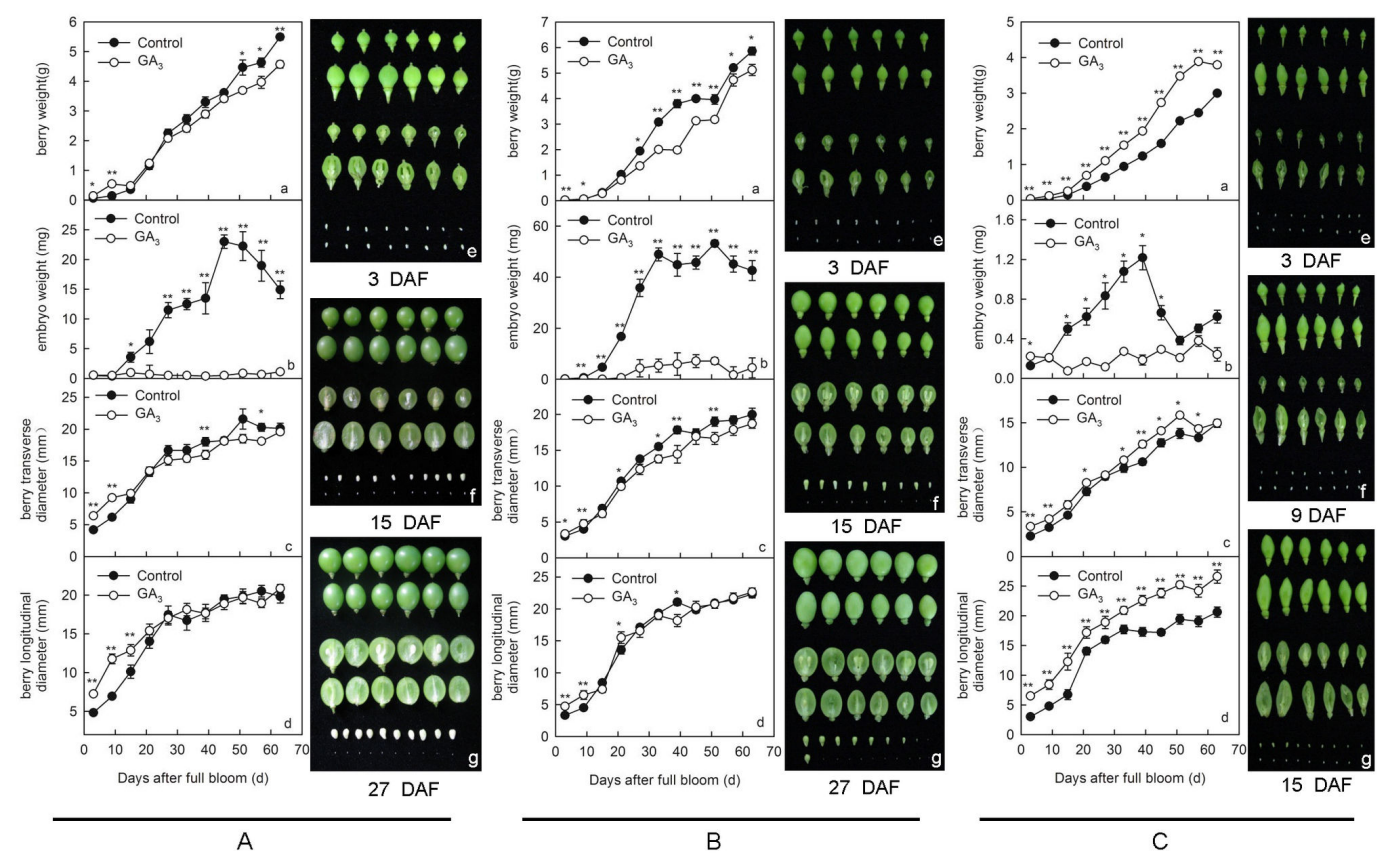
Effect of GA_3_ treatment on the shape and size of ‘Kyoho’, ‘Red Globe’ and ‘Thompson Seedless’ berries and seeds. Berry weight (a), seed weight (b), berry transverse diameter (c) and berry longitudinal diameter (d) in ‘Kyoho’ (A) and ‘Red Globe’ (B) cultivars. Photos are representative of untreated control and treated berries and seeds at 3 DAF (21 days after treatment in ‘Kyoho’ and 19 days after treatment in ‘Red Globe’) (e), 15 DAF (33 days after treatment in ‘Kyoho’ and 31 days after treatment in ‘Red Globe’) (f) and 27 DAF (45 days after treatment in ‘Kyoho’ and 43 days after treatment in ‘Red Globe’) (g); (C) Berry weight (a), seed weight (b), berry transverse diameter (c) and berry longitudinal diameter (d) in the ‘Thompson Seedless’ cultivar. Photos are representative of untreated control and treated berries and seeds at 3 DAF (19 days after treatment) (e), 9 DAF (25 days after treatment) (f) and 15 DAF (31 days after treatment) (g). Vertical bars indicate standard errors. Asterisks indicate significant differences between GA_3_ treated and untreated control samples (*P < 0.05, **P < 0.01, Student’s t–test). In each column, the first and second photos represent untreated control and GA_3_-treated berries, respectively, the third and fourth photos represent longitudinal sections of untreated control and GA_3_-treated berries, respectively, and the fifth and sixth photo represent untreated control and GA_3_-treated seeds, respectively.

To determine whether there were any morphological effects on either the berries or seeds following GA_3_ treatment, measurements (berry weight, transverse diameter, longitudinal diameter, as well as seed weight) were carried out 3, 9, 15, 21, 27, 33, 39, 45, 51, 57 and 63 d after full bloom (DAF) in the seeded cultivars ‘Kyoho’ and ‘Red Globe’, as well as the seedless ‘Thompson Seedless’ cultivar. As shown in Figure 1, patterns of change with respect to grape berry weight, transverse diameter, longitudinal diameter, and seed weight were similar in ‘Kyoho’ and ‘Red Globe’ cultivars following GA_3_ treatment, but differed in ‘Thompson Seedless’ plants. In the case of the ‘Kyoho’ cultivar, berry weight, transverse diameter and longitudinal diameter from GA_3_-treated plants were significantly higher than those of untreated plants from 3 to 9 DAF, with the difference in longitudinal diameter lasting until 15 DAF (Figure 1A a, c-g). Interestingly, ‘Kyoho’ berry weight and transverse diameter became significantly smaller than untreated berries after 15 DAF, while this same trend was observed after 21 DAF for longitudinal diameter (Figure 1A a, g). These results were very similar to those seen in ‘Red Globe’ berries (Figure 1B). Seed weight in GA_3_-treated ‘Kyoho’ plants was similar to that of untreated seeds from 3 to 9 DAF (Fig. 1A b, e), but from 15 DAF onwards, seeds from treated plants were found to weigh significantly less than those from untreated plants (Fig. 1A b, f-g). Similarly, seeds from GA_3_-treated ‘Red Globe’ plants were found to be significantly smaller than those harvested from untreated plants from 9 DAF onwards (Fig. 1B b, e-g). 

In contrast to ‘Kyoho’ and ‘Red Globe’ cultivars, the weight, transverse diameter and longitudinal diameter of berries from ‘Thompson Seedless’ plants treated with GA_3_ were always higher than those of untreated plants (Figure 1C a, c-g). Seeds from GA_3_-treated ‘Thompson Seedless’ plants weighed significantly more than seeds from untreated plants at 3 DAF, were similar in weight to untreated controls at 9 DAF, and weighed significantly less than untreated controls from 15 to 45 DAF. From 51 DAF onwards, GA_3_-treated ‘Thompson Seedless’ plants produced seeds that were slightly, but not significantly, lower in weight than untreated control seeds from the same cultivar (Fig. 1C b, e-g). Although the size of GA_3_-treated grape berries was different from that of untreated controls in all three cultivars, there was no effect on the content of soluble solids in grape berries after GA_3_ application (Figure S2).

### Seed development following GA_3_ application

**Figure 2:**
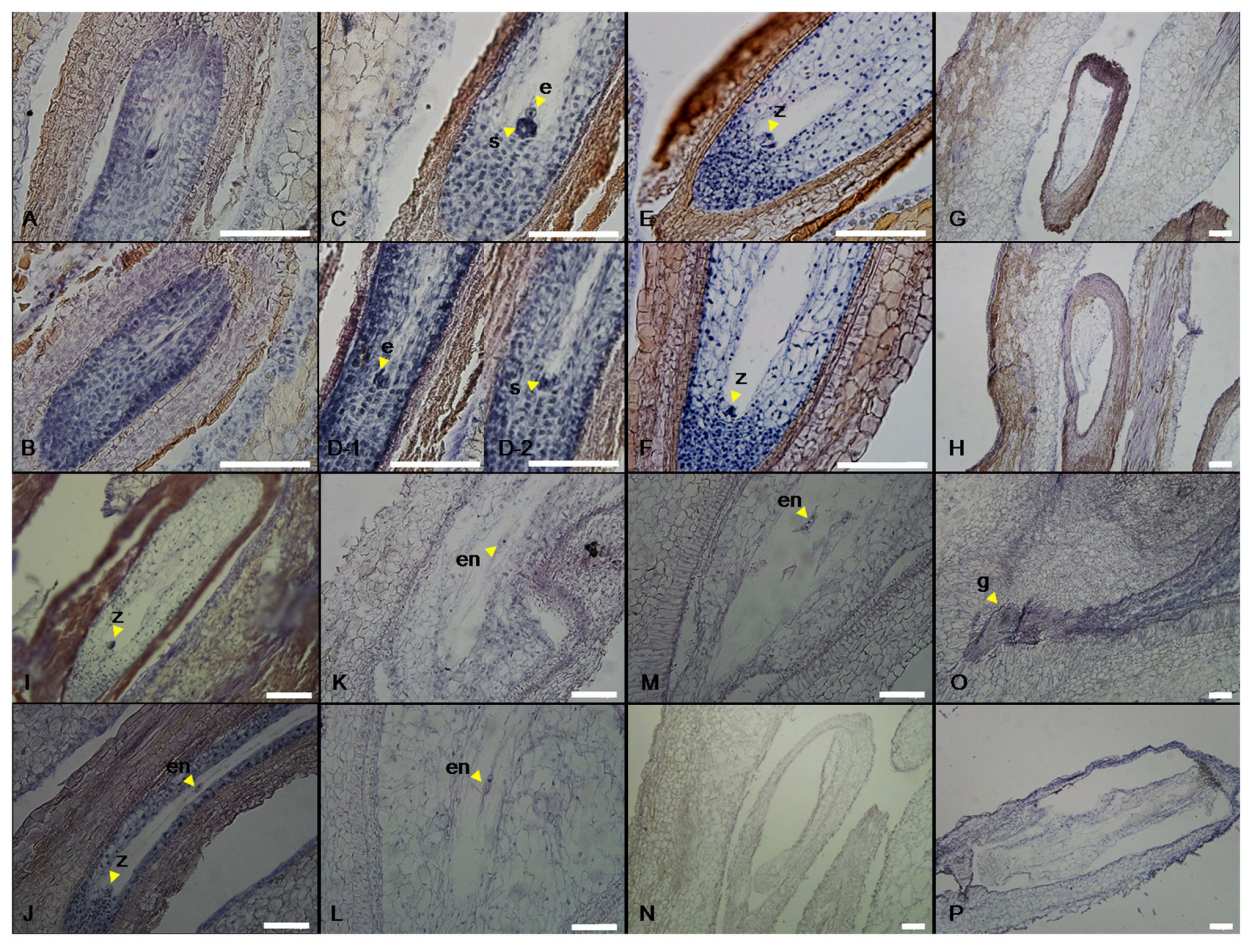
Seed development in untreated control and GA_3_-treated ‘Kyoho’ grape. (A and B) Normal embryo sacs with macrospore cells from untreated control (A) and treated (B) ovules 5 days after treatment; (C and D) egg cells and two synergids from untreated control (C) and treated (D) mature embryo sacs 9 days after treatment (D-1 and D-2 were contiguous sections); (E and F) zygotes from untreated control (E) and treated (F) seeds 17 days after treatment; (G and H) seeds with abnormal embryo sacs from treated grapes 17 days after treatment (G) and 21 days after treatment (3 DAF) (H); (I and J) normal zygotes from untreated control (I) and treated (J) seeds 21 days after treatment (3 DAF); (K and L) endosperm nuclei from untreated control (K) and treated (L) seeds 27 days after treatment (9 DAF); (M and N) embryo sac with endosperm nuclei from an untreated control seed (M) and abnormal embryo sac from a treated seed (N) 33 days after treatment (15 DAF); (O and P) early globular embryo from untreated control seed (O) and abnormal embryo sac from treated seed (P) 36 days after treatment (18 DAF). Scale bar = 100 μm, e: egg cell, en: endosperm nuclei, g: globular embryo, s: synergid, z: zygote.

**Figure 3:**
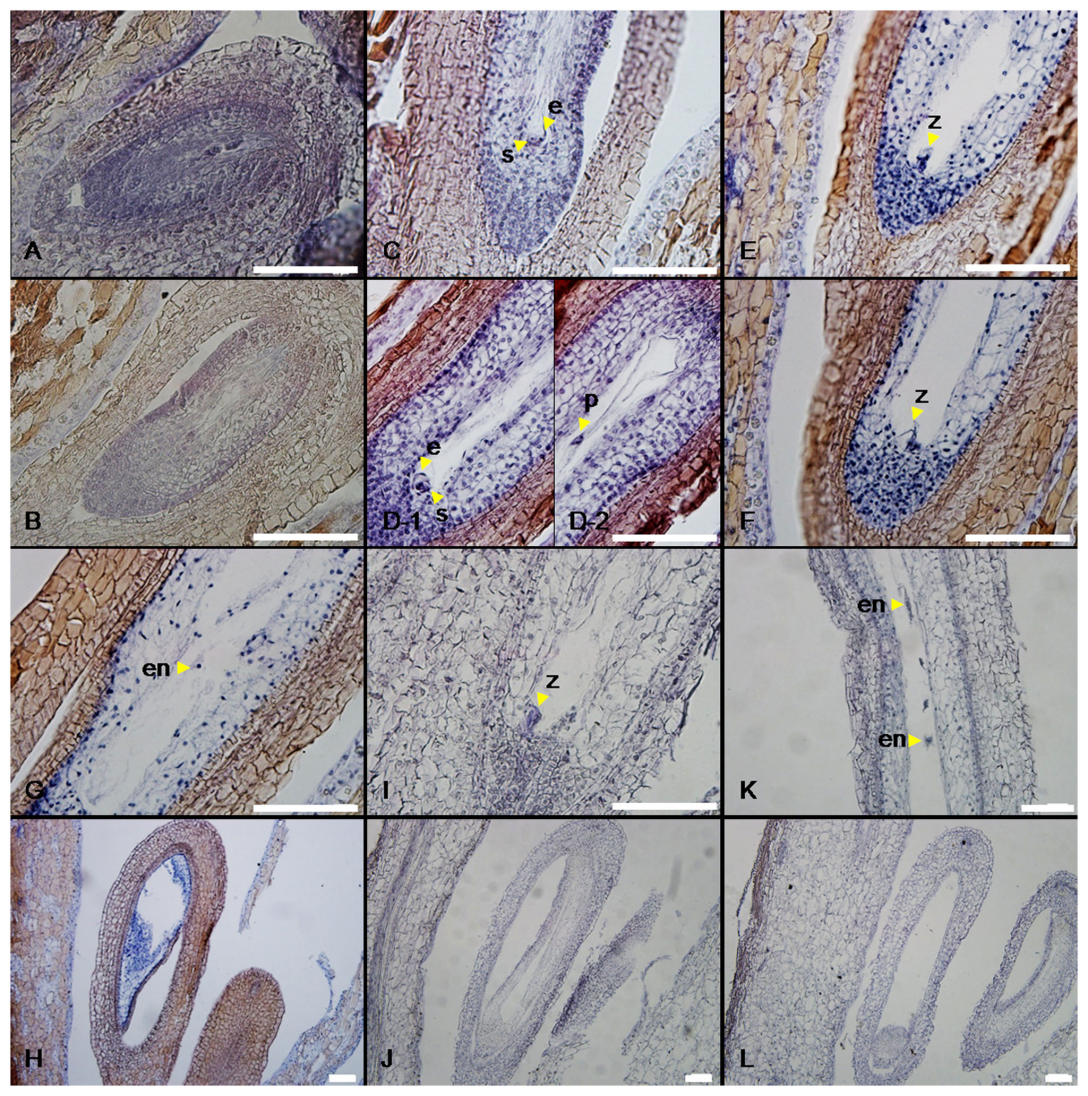
Seed development in untreated control and GA_3_-treated ‘Thompson Seedless’ grape. (A and B) Normal embryo sacs with macrospore cells from untreated control (A) and treated (B) ovules 5 days after treatment; (C and D) mitotic macrospore cell with premature embryo sac from untreated control (C) and an egg cell, two synergids and polar nucleus from treated ovule (D) 9 days after treatment (D-1 and D-2 were contiguous sections); (E and F) zygotes from untreated control (E) and treated (F) seeds 17 days after treatment (1 DAF); (G and H) abnormal endosperm nuclei from treated grape (G) and embryo with degrading embryo sac from treated grape (H) 17 days after treatment (1 DAF); (I and J) normal zygote from untreated control seed (I) and abnormal embryo sac from treated seed (J) 25 days after treatment (9 DAF); (K and L) endosperm nuclei from untreated control seed (K) and abnormal treated seed (L) 31 days after treatment (15 DAF). Scale bar = 100 μm, e: egg cell, en: endosperm nuclei, p: polar nucleus, s: synergid, z: zygote.

To determine the effects of GA_3_ treatment on seed development in grape, paraffin sections of GA_3_-treated and untreated ‘Kyoho’, ‘Red Globe’ and ‘Thompson Seedless’ flowers/seeds harvested every two days from 1 to 18 d following GA_3_ application, and subsequently every three days until 39 d after application, were stained with Ehrlich’s haematoxylin and observed using an optical microscope. The process of seed abortion among ‘Kyoho’, ‘Red Globe’ and ‘Thompson Seedless’ grapes following GA_3_ treatment was very similar (Figures 2-3; Figure S3). In the case of ‘Kyoho’ grape treated with GA_3_, normal seeds were evident up to 9 DAF (27 d after treatment) (Figure 2H-L), but from 15 DAF (33 d after treatment) onwards, GA_3_-treated seeds stopped growing and embryo sacs degenerated (Fig. 1Ab; Figure 2M-P). In the case of the ‘Red Globe’ cultivar, degenerated embryo sacs were observed in GA_3_-treated seeds 9 DAF (25 d after treatment) (Figure S3H), while abnormal zygotes and endosperm nuclei were also observed in some cases at this time point (Figure S3I-J). As was the case for ‘Kyoho’, all embryo sacs of GA_3_-treated ‘Red Globe’ seeds had degenerated by 15 DAF (31 d after treatment) (Figure S3K-L). In the ‘Thompson Seedless’ cultivar, normal zygotes, abnormal endosperm nuclei and the degeneration of embryo sacs were beginning to be apparent in GA_3_-treated seeds by 1 DAF (17 d after treatment), while the growth of GA_3_-treated seeds was completely halted and all embryo sacs had degenerated by 9 DAF (25 d after treatment) (Fig. 1Cb; Figure 3I-L). In order to assay the effects of GA_3_ treatment on anther development, paraffin sections of ‘Kyoho’, ‘Red Globe’ and ‘Thompson Seedless’ anthers were generated and it was found that there was no apparent difference between those treated with GA_3_ and control untreated anthers in any of the three cultivars (Figure S4).

### Changes in H_2_O_2_ and MDA content following GA_3_ treatment

It has been proposed that ROS, such as H_2_O_2_, play two very opposing roles in living organisms: at low levels they act as a signaling pathway to activate defense responses, while at high levels they exacerbate stress damage and signal programmed cell death [21]. One manner in which ROS can cause cell damage is through the lipid peroxidation pathway [22]. Since MDA content is a direct measure of lipid peroxidation, it was utilized as a marker to assess the degree of cell damage [23]. Therefore, we analyzed H_2_O_2_ accumulation and MDA content in all three grape cultivars following GA_3_ treatment from 1 to 75 d following application for the ‘Kyoho’ cultivar and 1 to 73 d following application for the ‘Red Globe’ and ‘Thompson Seedless’ cultivars. 

**Figure 4:**
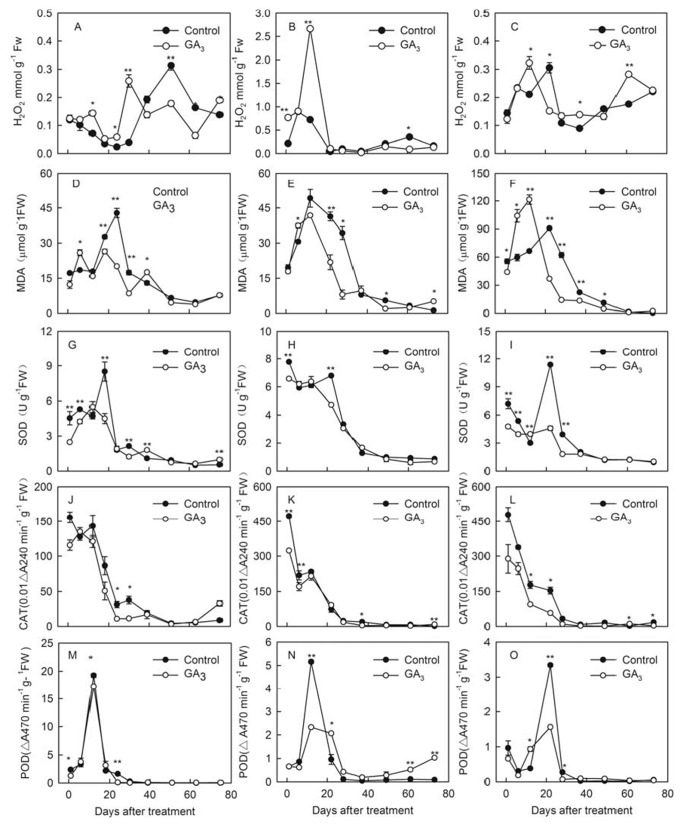
Changes in H_2_O_2_ and MDA accumulation, as well as antioxidant enzyme activities, after GA_3_ treatment. (A-C) H_2_O_2_ accumulation in ‘Kyoho’ (A), ‘Red Globe’ (B) and ‘Thompson Seedless’ (C) cultivars; (D-F) MDA accumulation in ‘Kyoho’ (D), ‘Red Globe’ (E) and ‘Thompson Seedless’ (F) cultivars; (G-I) SOD activity in ‘Kyoho’ (G), ‘Red Globe’ (H) and ‘Thompson Seedless’ (I) cultivars; (J-K) CAT activity in ‘Kyoho’ (J); ‘Red Globe’ (K) and ‘Thompson Seedless’ (L) cultivars; (M-O) POD activity in ‘Kyoho’ (M), ‘Red Globe’ (N) and ‘Thompson Seedless’ (O) cultivars. Data points represent the mean ± SE of at least three replicates. Asterisks indicate significant differences between GA_3_-treated and untreated control samples from the same cultivar (* P < 0.05, ** P < 0.01, Student’s t –test).

As shown in Figure 4, peaks in the accumulation of H_2_O_2_ were noted 12 d after GA_3_ treatment in ‘Kyoho’ (Figure 4A), ‘Red Globe’ (Figure B) and ‘Thompson Seedless’ (Figure 4C) berries. A second, larger peak was also observed in ‘Kyoho’ berries 30 d after treatment (12 DAF). These time points of peak H_2_O_2_ accumulation in treated samples differed substantially from patterns exhibited by untreated control samples, and occurred prior to the observed degeneration of embryo sacs in each case (15 DAF in ‘Kyoho’ and ‘Red Globe’ cultivars, 9 DAF in the ‘Thompson seedless’ cultivar, Figures 2-3, Figure S3, Figure 4A-C). 

Changes in MDA content in ‘Kyoho’ (Figure 4D), ‘Red Globe’ (Figure 4E) and ‘Thompson Seedless’ berries at various time points following GA_3_ treatment (Figure 4F) were similar in pattern to those observed with respect to H_2_O_2_ accumulation. In ‘Kyoho’ berries, peaks in MDA content were observed 6 d, 18 d and 39 d after GA_3_ treatment. Conversely, MDA content in both ‘Red Globe’ and ‘Thompson Seedless’ berries peaked at 6 d following treatment, with levels declining thereafter. Interestingly, in all three cultivars, MDA content 6 d after treatment was always significantly higher than in the untreated control. 

### Changes in the activities of various antioxidant enzymes in response to GA_3_ treatment

In order to prevent damage caused by high levels of ROS, cells must maintain a state of redox homeostasis. It has been suggested that this may be attained through interactions that occur between ROS and scavenger antioxidant enzymes. We analyzed the activities of three such enzymes (SOD, CAT and POD) in flowers/berries harvested between 1 and 75 d following GA_3_ treatment for the ‘Kyoho’ cultivar or 1 and 73 d following GA_3_ treatment for ‘Red Globe’ and ‘Thompson Seedless’ cultivars. In all cases, their activities were found to differ between treated and untreated controls (Figure 4G-O). Changes in the activities of SOD, CAT and POD in ‘Kyoho’ (Figure 4G, J, M), ‘Red Globe’ (Figure 4H, K, N) and ‘Thompson Seedless’ (Figure 4I, L, O) following treatment exhibited similar patterns, respectively. In all three cultivars, the activities of SOD from GA_3_-treated samples 1 d following treatment were significantly lower than those of untreated controls (Figure 4G-I). Treated samples also differed from untreated controls in that they lacked the distinct peaks in activity which were apparent 18 d after mock treatment in the case of ‘Kyoho’ (at full bloom) and 24 d after mock treatment (6 DAF) in the case of ‘Red Globe’ and ‘Thompson Seedless’. 

Similarly, the activity of CAT in the flowers of all three cultivars tested 1 d after GA_3_ treatment were diminished compared to untreated controls, however, this difference was significant only in the ‘Red Globe’ cultivar (Figure 4J-L). These values remained at least somewhat lowered in GA_3_-treated samples until 30 d following treatment in ‘Kyoho’, 12 d following treatment in ‘Red Globe’, and 22 d following treatment (6 DAF) in ‘Thompson Seedless’. 

In the case of POD activity, untreated control samples from all three cultivars exhibited a large peak 12 d after treatment in ‘Kyoho’ and ‘Red Globe’ berries, or 22 d after treatment (6 DAF) in ‘Thompson Seedless’ berries (Figure 4M-O). This peak was significantly reduced in GA_3_-treated samples from all three cultivars (Figure 4M-O).

**Figure 5:**
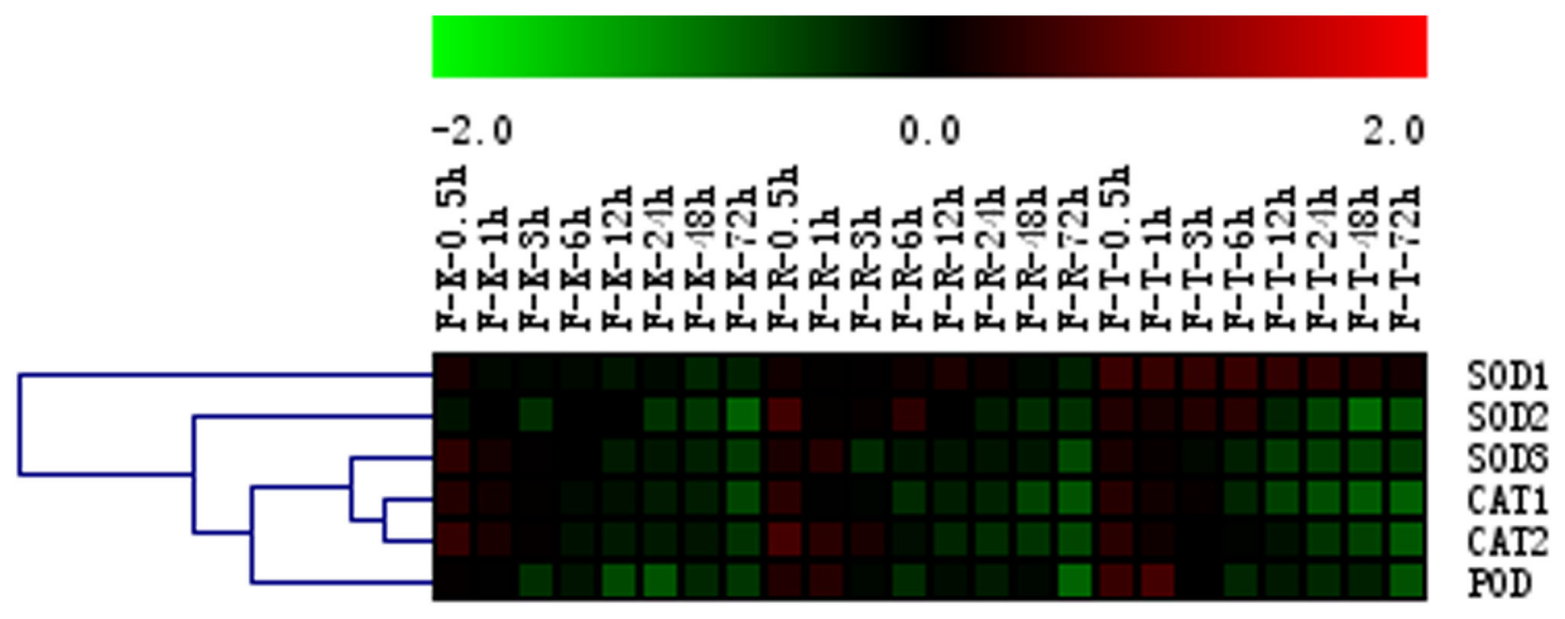
Hierarchical clustering of antioxidant gene expression in the flowers of three grape cultivars following GA_3_ treatment. Results of semi-quantitative RT-PCR assays were quantified using GeneTools software, and log-transformed values of the relative expression levels of genes encoding antioxidant enzymes following GA_3_ treatment compared to untreated controls were used for hierarchical cluster analysis. The color scale represents relative expression levels with red denoting up-regulation and green denoting down-regulation. Sampling times are indicated at the top of the figure; F represents floral tissue; K represents the ‘Kyoho’ cultivar; R represents the ‘Red Globe’ cultivar; T represents the ‘Thompson seedless’ cultivar.

In an effort to provide further evidence that changes were occurring with respect to SOD, CAT and POD in response to GA_3_ treatment in ‘Kyoho’, ‘Red Globe’ and ‘Thompson Seedless’ flowers, we analyzed the expression of their corresponding genes using semi-quantitative RT-PCR. As shown in Figure 5 (see also Figure S5), *SOD1* expression decreased in ‘Kyoho’ and ‘Red Globe’ flowers following GA_3_ application, while levels increased initially in the ‘Thompson Seedless’ cultivar. In the case of *SOD2*, expression appeared to decrease in ‘Kyoho’ flowers following treatment, while expression increased initially and then decreased following GA_3_ treatment, in ‘Red Globe’ and ‘Thompson Seedless’ flowers. *SOD3* transcript levels were increased 0.5 h and 1 h following treatment and declined thereafter in all three cultivars. 

Similar patterns were observed with the expression of catalase (*CAT1* and *CAT2*) and peroxidase genes (*POD*); *CAT1* and *CAT2* were both initially up-regulated and subsequently down-regulated in the flowers of all three cultivars, with a similar pattern being noted for *POD* expression in ‘Red Globe’ and ‘Thompson Seedless’ flowers. Conversely, *POD* expression in ‘Kyoho’ flowers simply appeared to be down-regulated beginning 3 h post-treatment (Figure 5, Figure S5). These results correlate well with the enzymatic activity results, whereby in all three cultivars SOD, CAT and POD activities in GA_3_-treated samples (with the exception of POD activity in ‘Red Globe’) tended to be reduced compared to untreated samples (Figure 4G-O). 

### Expression of genes related to seed development following GA_3_ treatment

**Figure 6:**
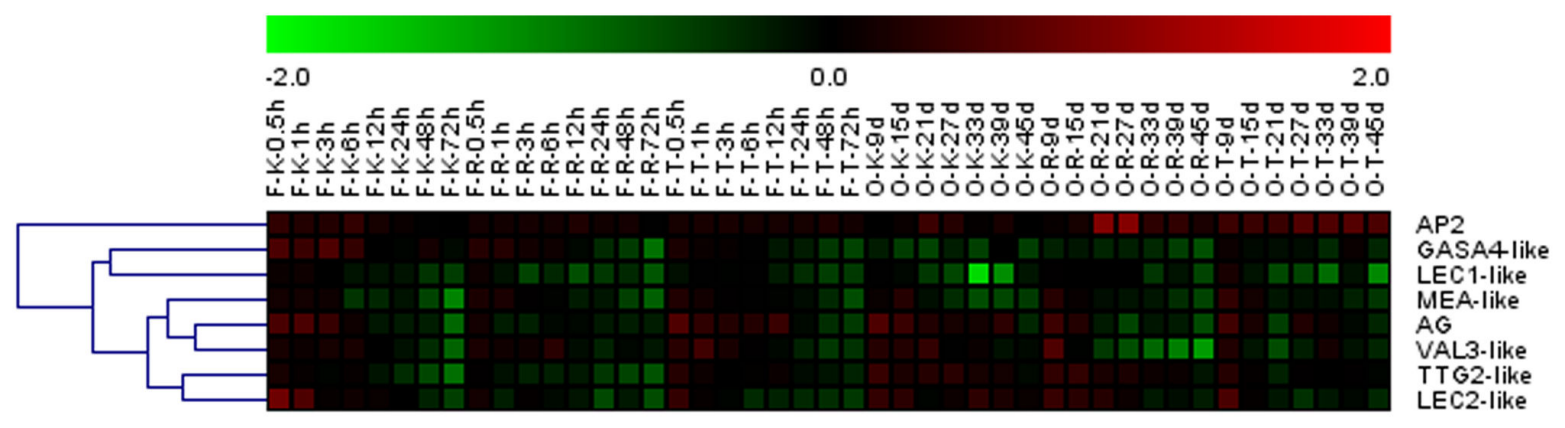
Hierarchical clustering of genes related to seed development in three grape cultivars following GA_3_ treatment. Results of semi-quantitative RT-PCR assays were quantified using GeneTools software, and log-transformed values of the relative expression levels of genes involved in seed development following GA_3_ treatments compared to untreated controls were used for hierarchical cluster analysis. The color scale represents relative expression levels with red denoting up-regulation and green denoting down-regulation. Sampling times are indicated at the top of the figure; F represents floral tissue; K represents the ‘Kyoho’ cultivar; S represents seed tissue; R represents the ‘Red Globe’ cultivar; T represents the ‘Thompson seedless’ cultivar.

To analyze the effect of GA_3_ application on grape genes that are putative orthologs of *Arabidopsis* genes known to be involved in seed development, we assayed the expression of MEA-like, *GASA4-*like, *AG*, *AP2*, *LEC1-*like, *LEC2*-like, *VAL3-*like and *TTG2*-like genes in flowers (at 0.5, 1, 3, 6, 12, 24, 48 and 72 h post-treatment) and seeds (at 9, 15, 21, 27, 33, 39 and 45 DAF, corresponding to 27, 33, 39, 45, 51, 57 and 63 d post-treatment in ‘Kyoho’ and 25, 31, 37, 43, 49, 55 and 61 d post-treatment in ‘Red Globe’ and ‘Thompson Seedless’) using semi-quantitative RT-PCR (Figure 6, Figure S6). In flowers, the expression patterns of *AG*, *LEC2*-like, and *TTG2*-like were similar in ‘Kyoho’ and ‘Thompson Seedless’ cultivars, whereby they appeared to be initially up-regulated and then subsequently down-regulated following treatment. This same pattern was observed for *VAL3*-like expression in the flowers of all three cultivars. Conversely, in ‘Red Globe’ flowers, the expression of *AG*, *LEC2*-like, and *TTG2*-like exhibited down-regulation post-GA_3_ treatment. In the seeds, these four genes all exhibited up-regulation in ‘Kyoho’ following treatment while in ‘Thompson Seedless’ they were all initially up-regulated and then subsequently down-regulated after GA_3_ application. In ‘Red Globe’ seeds, *AG* and *VAL3*-like were initially up-regulated and subsequently down-regulated while *TTG2*-like and *LEC2*-like were simply up-regulated following treatment. In the case of *AP2*, ‘Kyoho’ flowers, as well as seeds from all three cultivars, exhibited up-regulation following treatment, while both the flowers and seeds of all three cultivars displayed down-regulation of *LEC1*-like after GA_3_ application. MEA-like appeared to be down-regulated following treatment in the flowers of all three cultivars, while in the seeds, all three cultivars exhibited an initial up-regulation and subsequent down-regulation. In the case of *GASA4*-like, expression appeared to be up-regulated in ‘Kyoho’ flowers following treatment, while in ‘Red Globe’ and ‘Thompson Seedless’ flowers, as well as the seeds of all three cultivars, this gene was down-regulated post-treatment. 

## Discussion

Large fruit size, as well as reduced seed number and size, are major goals in the improvement of fruit crop species [1]. At present, exogenous GA_3_ application is often used as a strategy to induce seedlessness of seeded grapes; however, very little is known concerning the mechanism by which this occurs. Therefore, we endeavoured to investigate the effects of GA_3_ application before bloom on berry and seed development in two seeded (‘Kyoho’ and ‘Red Globe’) and one seedless (‘Thompson Seedless’) cultivars, as well as its effect on antioxidant enzyme activity and the expression of genes involved in seed development.

In terms of seed growth, GA_3_ application had a significant influence on seed abortion in ‘Kyoho’ and ‘Red Globe’ cultivars (Table 1, Figure 1A-B), and also affected the early stages of seed development in ‘Thompson Seedless’ grapes (Figure 1C). Exogenous application of GA_3_ is known to impair fertilization by either inducing seed abortion or reducing pollen tube growth in grapes and mandarin [14,17,20]. However, since our results demonstrated that seed growth in GA_3_-treated samples ceased after full bloom (Figure 1), it seems likely that in this case ovules were in fact being fertilized prior to seed abortion. This is also known to be the case in stenospermocarpic seedless grape cultivars, such as ‘Thompson Seedless’, whereby pollination and fertilization occur but the embryos are aborted [24]. Recently, it has been reported that embryo abortion in ‘Thompson Seedless’ began between 40-55 DAF [25]. However, seed abortion in GA_3_-treated samples of both seeded and seedless cultivars in the present study occurred between 9 and 15 DAF (Figures 1-3, Figure S3). These results demonstrate that exogenous GA_3_ application does not precisely imitate the natural process of embryo abortion that occurs in stenospermocarpic grapes. 

In order to determine whether fertilization did indeed occur prior to seed abortion following GA_3_ application, we analyzed the structural development of seeds in the three cultivars following treatment. In a similar fashion to untreated controls, ovule structure prior to pollination was not affected by GA_3_ application, and both zygotes and endosperm nuclei were observed in all three cultivars after full bloom following treatment (Figure 2, Figure 3, Figure S3), which suggests that the ovules had undergone fertilization normally. Subsequently, however, abnormal zygotes and endosperm nuclei became apparent, with complete abortion occurring 15 DAF in ‘Kyoho’ and ‘Red Globe’ cultivars (33 and 31 d after treatment, respectively), and 9 DAF (25 d after treatment) in the ‘Thompson Seedless’ cultivar following GA_3_ treatment (Figure 2, Figure 3, Figure S3). These results correspond well with the reduction in seed size noted in ‘Kyoho’ and ‘Red Globe’ cultivars at 15 DAF (33 and 31 d after treatment, respectively) (Figure 1) and suggest that instead of impairing fertilization, exogenous GA_3_ application is somehow interfering with seed development. 

Interestingly, GA_3_ treatment was found to have contrasting effects on mean berry weights of seeded and seedless cultivars, inhibiting growth in seeded ‘Kyoho’ and ‘Red Globe’ cultivars while stimulating growth in ‘Thompson Seedless’ (Figure 1). These results were consistent with previous findings in which growth was inhibited in the seeded grape cultivar ‘Emperador’ and promoted in the seedless cultivar ‘Emperatriz’ upon GA_3_ application [15]. 

Recently, it has been reported that reactive oxygen species (ROS), including superoxide anions and H_2_O_2_, act as regulators of growth and development in plants [26]. These molecules carry out their function through their interaction with, and damage caused to, substrates such as various enzymes and nucleic acids, serving as signaling molecules that induce cell death. Since the production of H_2_O_2_ has been found to be induced by GA [27], it seemed reasonable to suppose that GA_3_-induced seed abortion may result from the accumulation of ROS. Furthermore, since lipid peroxidation products can be utilized as biomarkers for oxidative injury, we also analyzed MDA content in flowers/berries to assess the amount of lipid peroxidation, and thus the degree of cell damage, following GA_3_ treatment [23]. In this study, H_2_O_2_ content in GA_3_-treated samples was significantly higher than that in untreated samples 12 d after GA_3_ treatment (in ‘Kyoho’, a second peak also occurred 30 d after GA_3_ treatment), while MDA content peaked 6 d after treatment, which in both cases was earlier than in untreated controls (Figure 4). The fact that H_2_O_2_ levels peaked after MDA upon GA_3_ treatment are consistent with previous findings that suggest that the accumulation of aldehydes (such as MDA) above a certain threshold level leads to the production of ROS (such as H_2_O_2_) [28,29].

Since excess levels of ROS cause damage, cells must attempt to maintain a state of redox homeostasis. One model of how this is attained suggests that interactions occur between ROS and antioxidant enzymes, which then act as a metabolic interface for signals derived from both metabolism and the environment. Indeed, it has been reported that the extent to which ROS accumulate is determined by the antioxidative system, which enables organisms to maintain proteins and other cellular components in an active state [30]. Interestingly, the antioxidant enzyme genes tested here (*SOD1*, *SOD2*, *SOD3*, *CAT1*, *CAT2* and *POD*) tended to be down-regulated in flowers after treatment with GA_3_ (Figure 5, Figure S5). In line with this, their corresponding enzyme activities (SOD, CAT, and POD) were reduced after GA_3_ application (Figure 4). This correlates well with previous findings whereby GA_3_ treatment was found to down-regulate the expression of *SOD*s [12] and decrease the activities of CAT and SOD [31]. All of the GA_3_-induced changes in H_2_O_2_ and MDA content, as well as antioxidant enzyme activities, occurred prior to GA_3_-induced seed abortion. 

It has been reported previously that hydroxyl radicals facilitate cell enlargement [32,33], and that the GA_3_-induced modulation of cell redox homeostasis may play a role in the determination of the shape and size of grape berries [12]. This could also be the case in this study, as we noted increased size of GA_3_-treated berries in all three cultivars prior to seed abortion (Figure 1) and during the same time frame as changes in H_2_O_2_ and MDA content, as well as antioxidant enzyme activities, were observed (Figure 4). Taken together, these results suggest that GA_3_ application interferes with redox homeostasis by decreasing the scavenging activity of ROS detoxification enzymes, thus leading to an accumulation of ROS and cell damage within seeds. 

To determine whether the observed changes in seed development upon GA_3_ application could also be due, at least in part, to variation in the expression of genes involved in seed development, we carried out semi-quantitative RT-PCR using flower and seed tissue and tested a selection of genes (Figure 6, Figure S6) that have been characterized previously in species such as *Arabidopsis*, *Petunia* and rice [34]. In *Arabidopsis*, the *MEDEA* (*MEA*) gene regulates cell proliferation in both the embryo and the endosperm by impacting gametophytic maternal control during seed development [35]. Mutations in this gene have been found to induce two distinct phenotypes: silique elongation without fertilization [36,37] or seed abortion [38]. In *Arabidopsis*, expression of the *MEA* gene was lower in the early stages of seed development and increased subsequently [39]. In this study, expression of the grape MEA-like gene was found to increase initially, then decline in GA_3_-treated flowers and seeds (Figure 6, Figure S6), which could be related to seed abortion in GA_3_-treated plants. 

*GASA4* regulates floral meristem identity and also positively influences both seed size and total seed yield [40]. It has been reported previously that the *GASA4* gene is up-regulated by GAs in meristematic regions and flower buds in *Arabidopsis*, but down-regulated in cotyledons and leaves [41]. In ‘Kyoho’ and ‘Red Globe’ seeds, we found the grape *GASA4-*like gene to be down-regulated following GA_3_ treatment (Figure 6, Figure S6). Similarly, *LEC1* is an important regulator of embryo development that activates the transcription of genes required for both embryo morphogenesis and cellular differentiation [42]. Moreover, *LEC1* RNA accumulates only during seed development in embryonic and endosperm tissue [42]. In grape, *LEC1*-like exhibited down-regulation in both flowers and seeds following GA_3_ treatment (Figure 6, Figure S6). Therefore, it is possible that the GA_3_-induced down-regulation of both *GASA4*-like and *LEC1*-like may be detrimental to seed development and contribute to GA_3_-induced seed abortion. Conversely, in *Arabidopsis*, mutation of the *AP2* gene causes the production of large seeds and an increase in both embryo cell number and cell size [43,44]. In grape, *AP2* expression was up-regulated by GA_3_ (Figure 6, Figure S6), which would likely interfere with embryo development and thus may have played an important role in GA_3_-induced seedlessness. 

In ‘Thompson Seedless’ and ‘Kyoho’ flowers and seeds, as well as ‘Red Globe’ seeds, the regulation of *LEC2*-like, *AG* and *TTG2*-like in response to GA_3_ was similar in that they tended to be initially up-regulated and then down-regulated following application. Since LEC1, which is an activator of seed development, is known to positively regulate LEC2 in many plants [45], we expected that *LEC2*-like expression would follow a similar pattern to *LEC1*-like in grape following GA_3_ treatment. As expected, the grape *LEC2-*like gene was down-regulated following GA_3_ treatment in flowers, which resembled our results for the *LEC1*-like gene (Figure 6, Figure S6). However, in ‘Kyoho’ and ‘Red Globe’ seeds, the response of *LEC2-*like expression to GA_3_ application did not correspond with *LEC1*-like as it was up-regulated (Figure 6, Figure S6). This up-regulation was also converse to the putative activating function of the grape *LEC2*-like gene, which suggests that this gene may play a slightly different role in grape [46]. 

Genes of the AGAMOUS (AG) subfamily have been shown to play crucial roles in reproductive organ identity, as well as fruit and seed development [47,48]. In agreement with our results for ‘Kyoho’ and ‘Thompson Seedless’ cultivars, it has been found previously that *AG* is up-regulated in inflorescence apices after GA treatment [49]. Furthermore, *TTG2*-like, which has been implicated in integument cell elongation and endosperm growth in *Arabidopsis* [50], was mainly down-regulated in floral tissues and up-regulated in seeds following GA_3_ application. Taken together, these expression results lend credence to the suggestion that GA_3_-induced seed abortion is a complex process, with changes in the expression of genes related to seed development playing a role.

In the present study, we investigated the effects of GA_3_ application before full bloom on berry and seed development in two seeded (‘Kyoho’ and ‘Red Globe’) and one seedless (‘Thompson Seedless’) cultivar, as well as its effect on ROS content and antioxidant enzyme activity, and the expression of genes involved in seed development. Our findings suggest that GA_3_-induced seed abortion is a result of abnormal seed development and may be caused, at least in part, by an impairment of redox homeostasis in flowers/berries resulting in oxidative damage to the seeds, as well as by changes in the expression of genes related to seed development. While these findings have enhanced our knowledge of how seedlessness is induced by exogenous GA_3_ application in grape, further analyses will be required to provide a more in depth understanding of the exact mechanism of GA_3_-induced seedlessness due to the apparent complexity of the mechanism involved. 

## Materials and Methods

### Plant Material and GA_3_ treatment

Seeded grape cultivars ‘Kyoho’ (*Vitis vinifera × V. labrusca*) and ‘Red Globe’ (*V. vinifera*), as well as the seedless ‘Thompson Seedless’ (*V. vinifera*) cultivar, were grown in an 8-year-old vineyard situated in an experimental field of Northwest A&F University, Yangling, Shaanxi, China (34° 20’ N, 108°24’ E). Both treated and untreated control experiments consisted of three biological replicates, with five vines per replicate, respectively. Fifteen clusters were kept on each vine, and treatment was carried out at 18 d before full bloom in ‘Kyoho’ and 16 d before full bloom in ‘Red Globe’ and ‘ Thompson Seedless’ cultivars. Initially, clusters were soaked in 0.05% Tween-20 (Roche, Basel, Switzerland) for 3 s to enhance subsequent uptake of the phytohormone. GA_3_ treatment was then carried out by soaking clusters in 100 mg L^-1^ GA_3_ (Sigma-Aldrich, St. Louis, MO, USA) dissolved in a small amount of 100% ethanol for 5 s (Figure S1) [13]. Untreated control clusters were subject to the same process without GA_3_.

### Berry and seed measurements

Berries and seeds were harvested for analyses of berry weight, transverse diameter, longitudinal diameter and seed weight 3, 9, 15, 21, 27, 33, 39, 45, 51, 57 and 63 d after full bloom (DAF, corresponding to 21, 27, 33, 39, 45, 51, 57, 63, 69, 75 and 81 d after treatment in ‘Kyoho’ and 19, 25, 31, 37, 43, 49, 55, 61, 67, 73 and 79 d after treatment in ‘Red Globe’ and ‘Thompson Seedless’). Fifty GA_3_-treated and untreated control berries, respectively, were randomly collected for the measurements at each time point, while one hundred GA_3_-treated and untreated control seeds, respectively, were randomly harvested for seed weight measurements.

### Analysis of seed development

For morphological analyses of developing seeds, flowers, young grape berries and seeds were collected one day after GA_3_ treatment, every two subsequent days for 18 d, and then every three days until 39 d after application. Three biological replicates were harvested in each case. Following fixation in Carnoy’s fluid (3:1 anhydrous alcohol/glacial acetic acid) for 12–24 h at room temperature, the samples were dehydrated through an ethanol series (30%, 50% and 70%) and stored in 70% ethanol at 4°C until further use [51]. Samples stored at 4°C were further dehydrated with a graded ethanol series (85%, 95% and 100%), infiltrated with xylene, and embedded in paraffin (Taiva, Hubei, China). Sections 8-10 μm in thickness were transferred onto poly-L-Lys-coated glass slides (WHB, Shanghai, China), deparaffinized with xylene, and re-hydrated through an ethanol series (100%, 95%, 85%, 70%, 50% and 30%). The resulting sections were stained with Ehrlich’s haematoxylin (Saichi, Shanghai, China) for 30 minutes at room temperature, dehydrated with an ethanol series, infiltrated with xylene, sealed with resinene (XT, Shanxi, China), and finally mounted beneath a coverslip. Slides were observed using an optical microscope (OLYMPUS BH-2, Japan) and photos were obtained using an attached digital camera (OLYMPUS DP72, Japan).

### Hydrogen peroxide (H_2_O_2_) and Malondialdehyde (MDA) Determination

In the case of the ‘Kyoho’ cultivar, flowers/berries were harvested 1, 6, 12, 18, 24 (6 DAF), 30 (12 DAF), 39 (21 DAF), 51 (33 DAF), 63 (45 DAF) and 75 (57 DAF) d after treatment, while tissues from ‘Red Globe’ and ‘Thompson Seedless’ cultivars were harvested 1, 6, 12, 22 (6 DAF), 28 (12 DAF), 37 (21 DAF), 49 (33 DAF), 61 (45 DAF) and 73 (57 DAF) d after treatment. H_2_O_2_ was extracted from the harvested tissue and levels were determined as described previously [52]. MDA accumulation was assayed in the same tissue samples using the thiobarbituric acid test [53].

### Antioxidant Enzyme Assay

Homogenization of 0.5 g GA_3_-treated and untreated flowers/berries harvested at the same time points described in the previous section was carried out in 5 mL of 50 mM sodium phosphate buffer (pH 7.0) containing 1 mM ethylenediaminetetraacetic acid (EDTA) (Taiva) and 5% soluble polyvinyl pyrrolidone (Sigma-Aldrich, St. Louis, MO, USA). The resulting homogenate was centrifuged at 13,000 × g for 30 min at 4°C and the supernatant was utilized for subsequent measurement of superoxide dismutase (SOD, EC 1.15.1.1), catalase (CAT, EC 1.11.1.6) and peroxidase (POD, EC 1.11.1.7) activities.

Total SOD activity was assayed by monitoring the inhibition of the photochemical reduction of nitro blue tetrazolium (NBT; Amresco, Solon, OH, USA), which produces a blue colouration in the presence of reactive oxygen, using a UV-1700 UV-Vis Spectrophotometer (Shimadzu, Kyoto,  Japan) as described previously [54]. In brief, 3 ml reaction mixtures comprising 50 mM sodium phosphate buffer (pH 7.0), 13 mM methionine, 75 μM NBT, 2 μM riboflavin (Amresco), 0.1 mM EDTA, and 100 μl enzyme extract were illuminated for 20 min at an intensity of 4000 lx using a GXZ-280B illumination incubator (Ningbo Jiangnan Instrument Factory, Ningbo, China) and then assayed at 560 nm. One unit of SOD activity was defined as the amount of enzyme required to cause 50% inhibition of NBT reduction at 560 nm. 

CAT activity was determined by monitoring the consumption of H_2_O_2_ at 240 nm for 3 min as described previously [55]. In each case, reaction mixtures comprised 50 mM sodium phosphate buffer (pH 7.0), 10 mM H_2_O_2_ and 200 μl of enzyme extract in a 3 ml volume. CAT activity was reported as 0.01 times the amount of change in absorption per min per g FW. 

The activity of POD was assayed by adding 100 μl aliquots of tissue extracts to 3 ml of assay solution, consisting of 13 mM guaiacol (Amresco), 5 mM H_2_O_2_ and 50 mM sodium phosphate buffer (pH 7.0) [56]. Increases in optical density were then measured at 470 nm for 3 min at 25°C using a UV-1700 UV-Vis Spectrophotometer (Shimadzu). POD activity was expressed as the change in absorbance per min per g FW. 

### Gene expression analysis by semi-quantitative RT-PCR

Total RNA was extracted from GA_3_-treated and untreated ‘Kyoho’, ‘Red Globe’ and ‘Thompson Seedless’ flowers collected at 0.5, 1, 3, 6, 12, 24, 48 and 72 h post-treatment, as well as seeds harvested at 9, 15, 21, 27, 33, 39 and 45 DAF (corresponding to 27, 33, 39, 45, 51, 57 and 63 d after treatment in ‘Kyoho’ and 25, 31, 37, 43, 49, 55 and 61 d after treatment in ‘Red Globe’ and ‘Thompson Seedless’), using the E.Z.N.A. ^®^ Plant RNA Kit according to the manufacturer’s instructions (Omega Biotek, Norcross, GA, USA). First-strand cDNA was synthesized using 500 ng total RNA as template along with PrimeScript TM RTase and an oligo dT primer (TaKaRa Biotechnology, Dalian, China). Subsequent PCR assays were carried out in a final volume of 20 µl including 1µl of cDNA template, 0.4 μmol L^-1^ gene-specific primers, and 2x HotMaster *Taq* PCR MasterMix (Tiangen Biotech Co. Ltd., Beijing, China). The sequences of the gene-specific primers utilized in this study can be found in Table S1, with the exception of those used for amplification of the grape *CAT1*, *SOD1*, *SOD2* and *SOD3* genes, which have been described previously [57]. Since several of the genes tested had not previously been identified in grape, the amino acid sequences of *Arabidopsis GASA4* (CAA66909.1), *LEC1* (NP_173616.2), *LEC2* (NP_564304.1), *MEA* (NP_563658.1), *TTG2* (NP_001078015.1), and *VAL3* (NP_193886.2) were used as query sequences to search for putative orthologs in the grape genome database, GENOSCOPE (http://www.genoscope.cns.fr/spip/), as well as the National Center for Biotechnology Information (NCBI, http://www.ncbi.nlm.nih.gov). The grape *Actin1* gene fragment (GenBank Accession number AY680701) was amplified with primers F (5’-GAT TCT GGT GAT GGT GTG AGT-3’) and R (5’-GAC AAT TTC CCG TTC AGC AGT-3’) as an internal standard. Thermal parameters for amplification were as follows: 94°C for 3 min, 25 cycles of 94°C for 30 s, 58°C for 30 s and 72°C for 30 s, followed by a final elongation at 72°C for 5 min. Three independent biological replicates and technical replicates, respectively, were performed in each case. Subsequently, 10 μl of the resulting PCR products were separated on a 1.2% (w/v) agarose gel, stained with ethidium bromide and photographed under UV illumination. The results of all semi-quantitative RT-PCR assays were quantified using GeneTools software (Syngene, Frederick, MD, USA) and log-transformed values of relative expression levels following GA_3_ treatment compared to untreated controls were used for hierarchical cluster analysis with the MultiExperiment Viewer (MeV version 4.8.1, http://www.tm4.org/). 

### Statistical analysis

Data were analyzed using student’s t-tests carried out using SPSS software (SPSS 17.0^®^, Chicago, IL, USA).

## Supporting Information

Table S1 **Primers used for semi-quantitative RT-PCR experiments.**
(DOC)

Figure S1 **Inflorescences of three grape cultivars.** (A) Inflorescence from the ‘Kyoho’ cultivar 18 days before full bloom; (B and C) Inflorescences from ‘Red Globe’ (B) and ‘Thompson Seedless’ (C) cultivars 16 days before full bloom.
(TIFF)

Figure S2 **Soluble solid content of control and GA_3_-treated mature grape berries.** Fifty berries were randomly selected for measurement. Vertical bars indicate standard errors.
(TIF)

Figure S3 **Seed development in untreated control and GA_3_-treated ‘Red Globe’ grapes.** (A and B) Normal embryo sacs with macrospore cells from untreated control (A) and treated (B) ovules 5 days after treatment; (C and D) egg cells and two synergids from untreated control (C) and treated (D) embryo sacs 9 days after treatment; (E and F) zygotes from untreated control (E) and treated (F) seeds 17 days after treatment (1 DAF); (G and H) seeds with abnormal embryo sacs from treated grape 17 days after treatment (1 DAF) (G) and 25 days after treatment (9 DAF) (H); (I and J) normal zygote from an untreated control seed (I) and abnormal zygote and endosperm nuclei from a treated seed (J) 25 days after treatment (9 DAF) (J-1 and J-2 were contiguous sections); (K and L) normal untreated control seed (K) and abnormal treated seed (L) 31 days after treatment (15 DAF). Scale bar = 200 μm, e: egg cell, en: endosperm nuclei, s: synergid, z: zygote.
(TIF)

Figure S4 **Anthers from ‘Kyoho’, ‘Red Globe’ and** **‘Thompson Seedless’ cultivars 5 days after GA_3_ treatment**.
(A-B) ‘Kyoho’ anthers; (C-D) ‘Red Globe’ anthers; (E-F) ‘Thompson Seedless’ anthers. Scale bar = 400 μm.
(TIFF)

Figure S5 **Semi-quantitative RT-PCR analysis of the expression of genes encoding antioxidant enzymes in ‘Kyoho’, ‘Red Globe’ and ‘Thompson Seedless’ flowers following GA_3_ treatment.** For each gene, the bands in the top row represent amplified products from flowers of untreated controls while bands in the bottom row represent amplified products from flowers of GA_3_-treated samples.
(TIFF)

Figure S6 **Semi-quantitative RT-PCR analysis of the expression of genes related to seed development in ‘Kyoho’, ‘Red Globe’ and ‘Thompson Seedless’ flowers (**A**) and seeds (**B**) following GA_3_ treatment.** For each gene, bands in the top row represent amplified products from flowers and seeds of untreated control samples while bands in the bottom row represent amplified products from flowers and seeds of GA_3_-treated samples.
(TIFF)
