# Supplementary material for: Effect of GA3 Treatment on Seed Development and Seed-Related Gene Expression in Grape
Source: PLoS One. 2013 Nov 5;8(11):e80044. doi: 10.1371/journal.pone.0080044 (PMC3818301; doi:10.1371/journal.pone.0080044)
Supplement: Table S1 — Primers used for semi-quantitative RT-PCR experiments. (DOC) [file pone.0080044.s001.doc]

| **Genes** | **Locus (GENOSCOPE)** | **Forward primer (5'–3')** | **Reverse primers ( 5'–3')** |
| --- | --- | --- | --- |
| *AP2* | GSVIVT01022081001 | AGCGGCATTAAAGCCAGGTG | ACTGCTGCTTGGAAACTGAATCTG |
| *GASA4-like* | [GSVIVT01011412001](http://www.quantprime.de/minimal.php?page=transcripts&subaction=geneinfo&identifier=GSVIVT01011412001&organismid=622) | TGTTGTAGGCCTCTTATGGTCCTG | GCCACTCTCATCCAAGGGACAAAC |
| *LEC1-like* | GSVIVT01002895001 | GGAGTGTGTGTCTGAGTACG | CCCAAACTCCACATTCCTCTT |
| *MEA-like* | GSVIVT01028124001 | GGAGAGGCGATGCAGATTATAC | CTTTGACAGGGGCATTCCTT |
| *VAL3-like* | GSVIVT01003212001 | ATGAGGGAAGGTTCTGTGGG | GGCAGTATCCAACAACGTGAA |
| *LEC2-like* | [GSVIVT01034419001](http://www.quantprime.de/minimal.php?page=transcripts&subaction=geneinfo&identifier=GSVIVT01034419001&organismid=622) | GCATTGAAGTGGCTAAACGG | GGCATCCATACTCAGACGAAG |
| *AG* | GSVIVT01021303001 | TTGTGGGGAGATTAGTGTAAAGTG | TTGCTGGCTGGTGAGGAAC |
| *TTG2-like* | GSVIVT01025562001 | TTCTGGGTGATGGCTTTCG | CTGGGTCCTCTGATGTCT |
| *CAT1*^a^ | GSVIVT00004081001 | TGCGGAGACATTCCCTATTC | TGTTGATGAAACGCTCTTGC |
| *CAT2* | GSVIVT00002880001 | ATCTGGATCTCCTACTGGTCTC | TTGGCTTGCTCTCTGTAACTC |
| *SOD1*^a^ | GSVIVT00029451001 | GGCGATTCATCTACGGTTGT | CAACCCAGTGAACCTTTTGG |
| *SOD2* ^a^ | GSVIVT00014163001 | CATGTTCAAGGAAGGGCAAT | CCCAATGGTATTCCAACGTC |
| *SOD3* ^a^ | GSVIVT00008877001 | AATGAGGGTGCTTGTGGAAC | AGGCCAGAAAGACTCCCAGT |
| *POD* | GSVIVT01032513001 | GGATCGTCATTACCCAGAGATG | CACGAAGATAGAGTGGGACAAG |

Table S1 Primers used for semi-quantitative RT-PCR experiments

^a^ primers were cited .

1. Vergara R, Parada F, Rubio S, Pérez FJ (2012) Hypoxia induces H_2_O_2_ production and activates antioxidant defence system in grapevine buds through mediation of H_2_O_2_ and ethylene. Journal of Experimental Botany 63: 4123-4131.
