# Supplementary figures and images for: Effect of GA3 Treatment on Seed Development and Seed-Related Gene Expression in Grape
Source: PLoS One. 2013 Nov 5;8(11):e80044. doi: 10.1371/journal.pone.0080044 (PMC3818301; doi:10.1371/journal.pone.0080044)

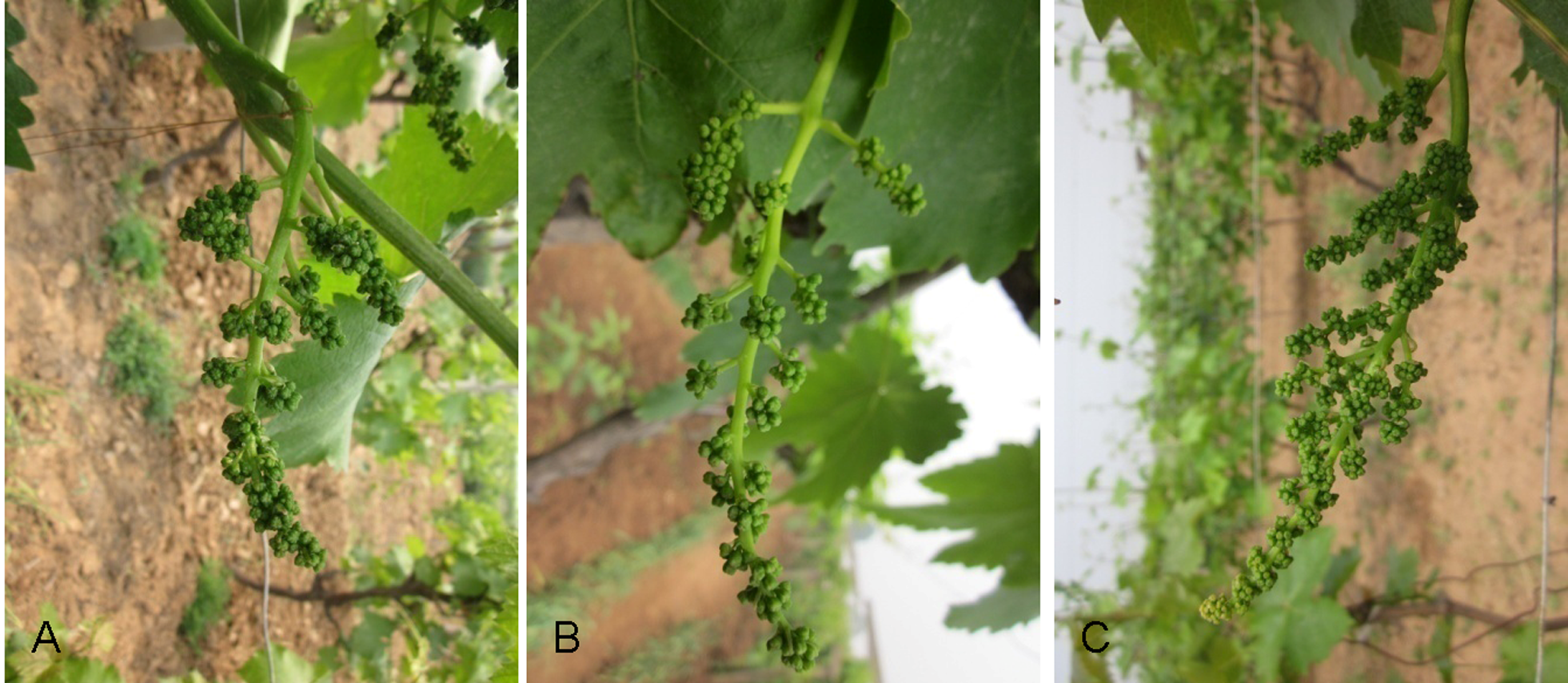

Supplement: Figure S1 — Inflorescences of three grape cultivars. (A) Inflorescence from the ‘Kyoho’ cultivar 18 days before full bloom; (B and C) Inflorescences from ‘Red Globe’ (B) and ‘Thompson Seedless’ (C) cultivars 16 days before full bloom. (TIFF) [file pone.0080044.s002.tiff]

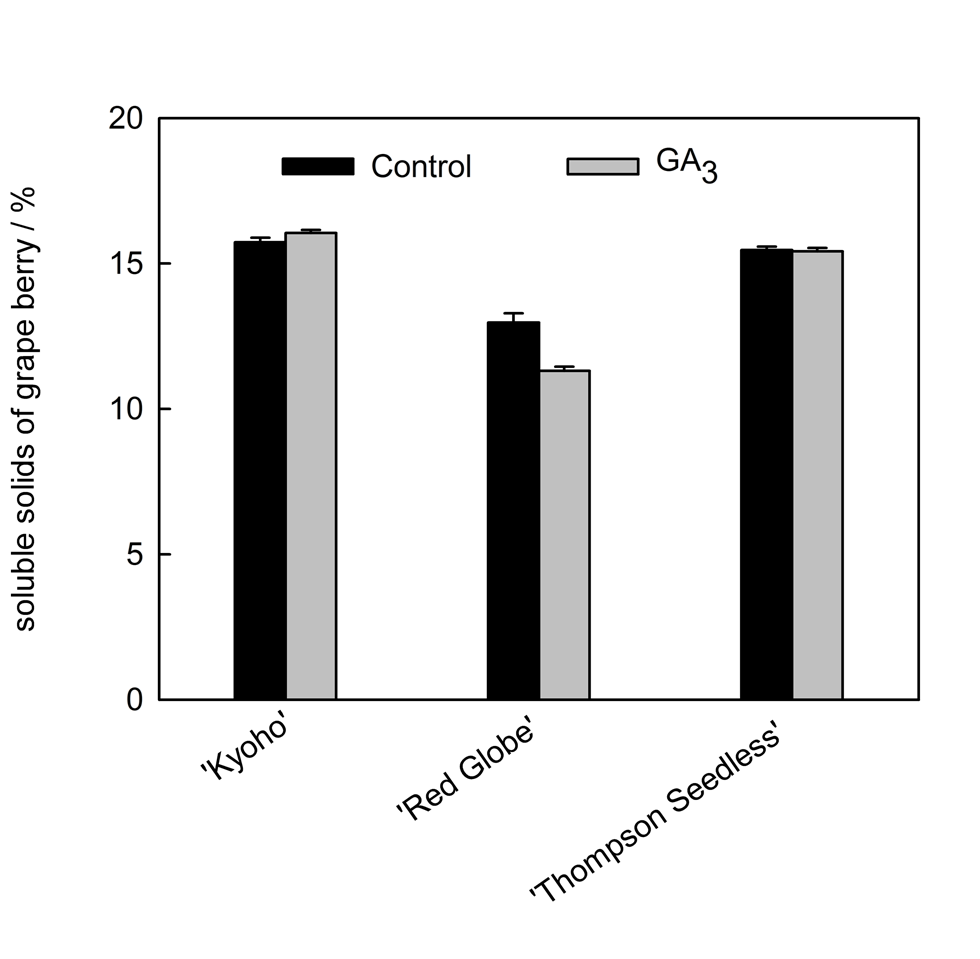

Supplement: Figure S2 — Soluble solid content of control and GA3-treated mature grape berries. Fifty berries were randomly selected for measurement. Vertical bars indicate standard errors. (TIF) [file pone.0080044.s003.tif]

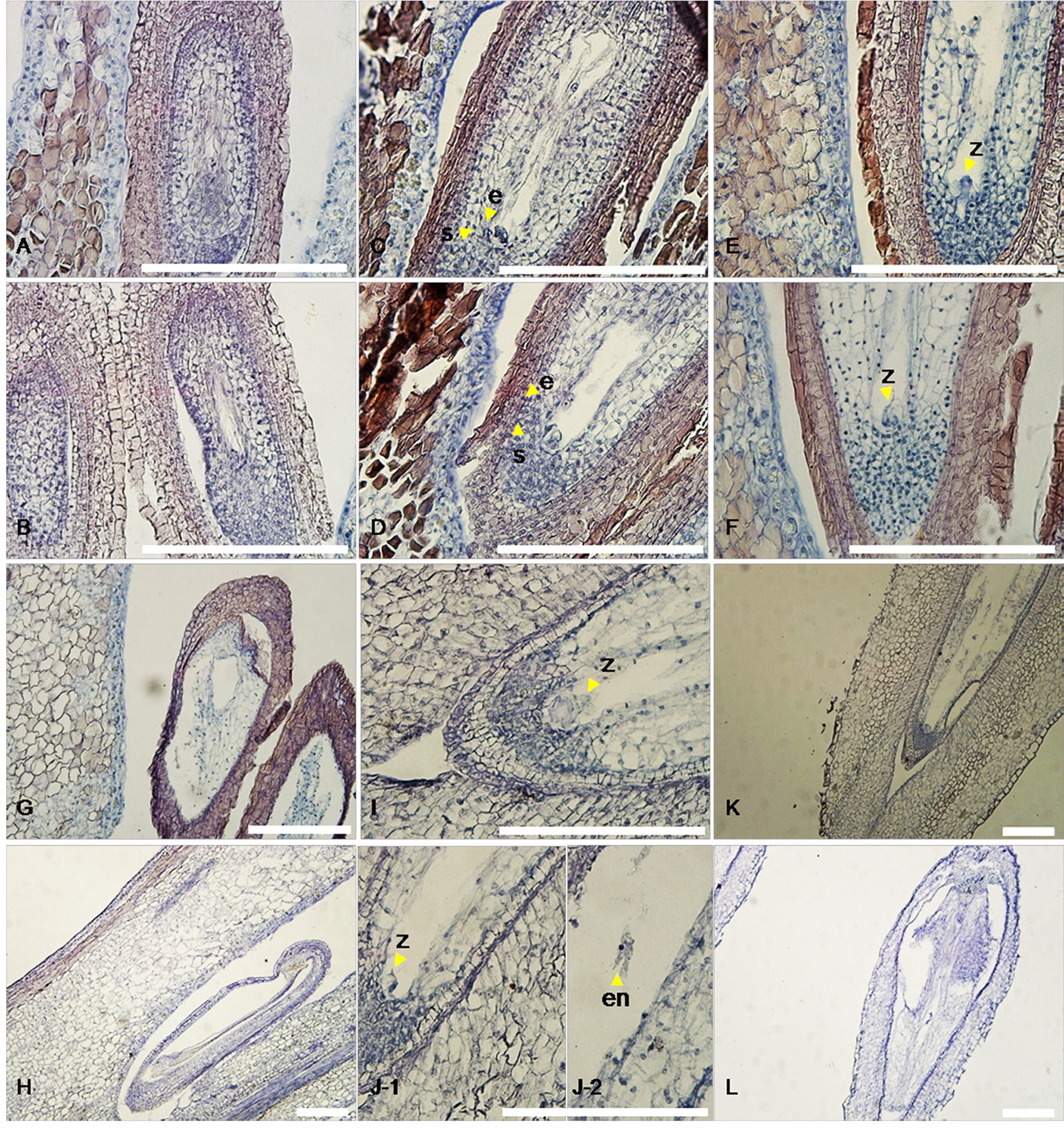

Supplement: Figure S3 — Seed development in untreated control and GA3-treated ‘Red Globe’ grapes. (A and B) Normal embryo sacs with macrospore cells from untreated control (A) and treated (B) ovules 5 days after treatment; (C and D) egg cells and two synergids from untreated control (C) and treated (D) embryo sacs 9 days after treatment; (E and F) zygotes from untreated control (E) and treated (F) seeds 17 days after treatment (1 DAF); (G and H) seeds with abnormal embryo sacs from treated grape 17 days after treatment (1 DAF) (G) and 25 days after treatment (9 DAF) (H); (I and J) normal zygote from an untreated control seed (I) and abnormal zygote and endosperm nuclei from a treated seed (J) 25 days after treatment (9 DAF) (J-1 and J-2 were contiguous sections); (K and L) normal untreated control seed (K) and abnormal treated seed (L) 31 days after treatment (15 DAF). Scale bar = 200 μm, e: egg cell, en: endosperm nuclei, s: synergid, z: zygote. (TIF) [file pone.0080044.s004.tif]

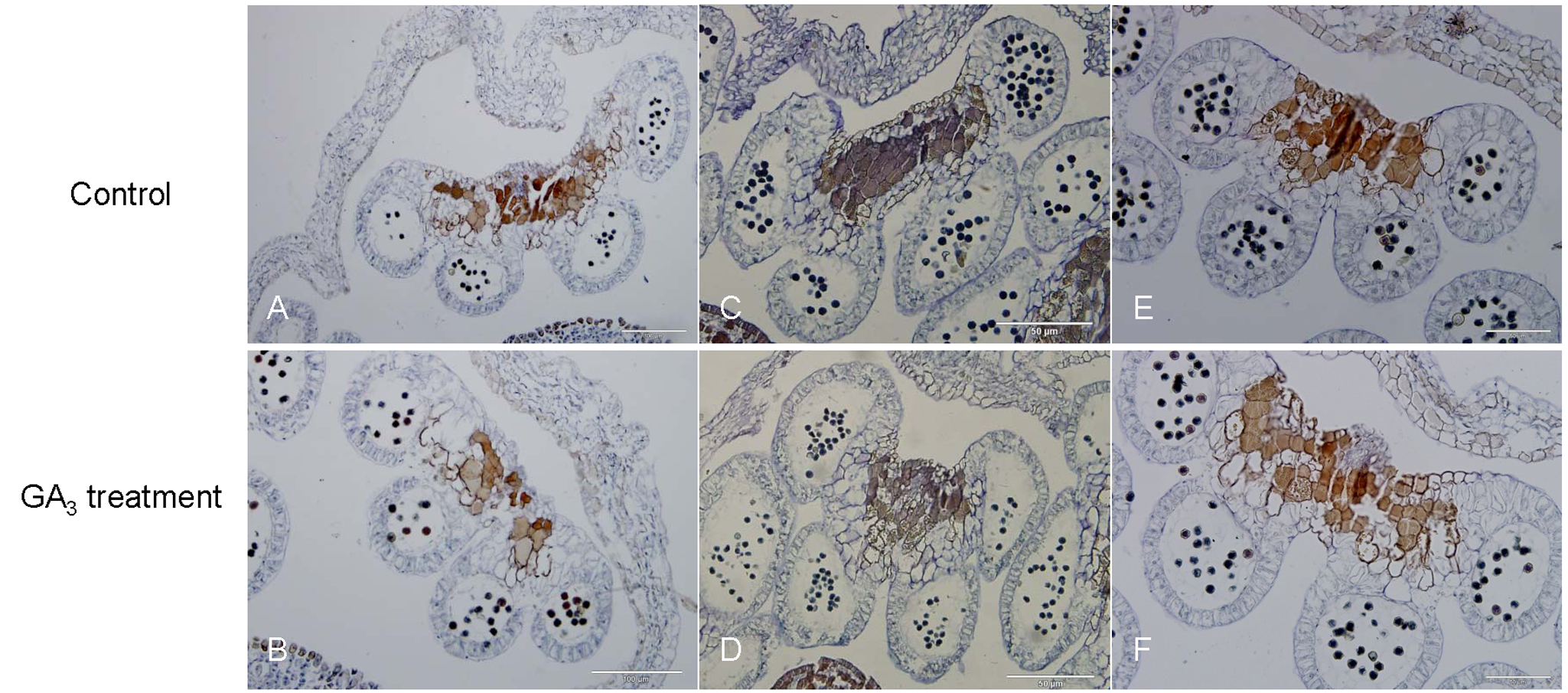

Supplement: Figure S4 — Anthers from ‘Kyoho’, ‘Red Globe’ and ‘Thompson Seedless’ cultivars 5 days after GA3 treatment. (A-B) ‘Kyoho’ anthers; (C-D) ‘Red Globe’ anthers; (E-F) ‘Thompson Seedless’ anthers. Scale bar = 400 μm. (TIFF) [file pone.0080044.s005.tiff]

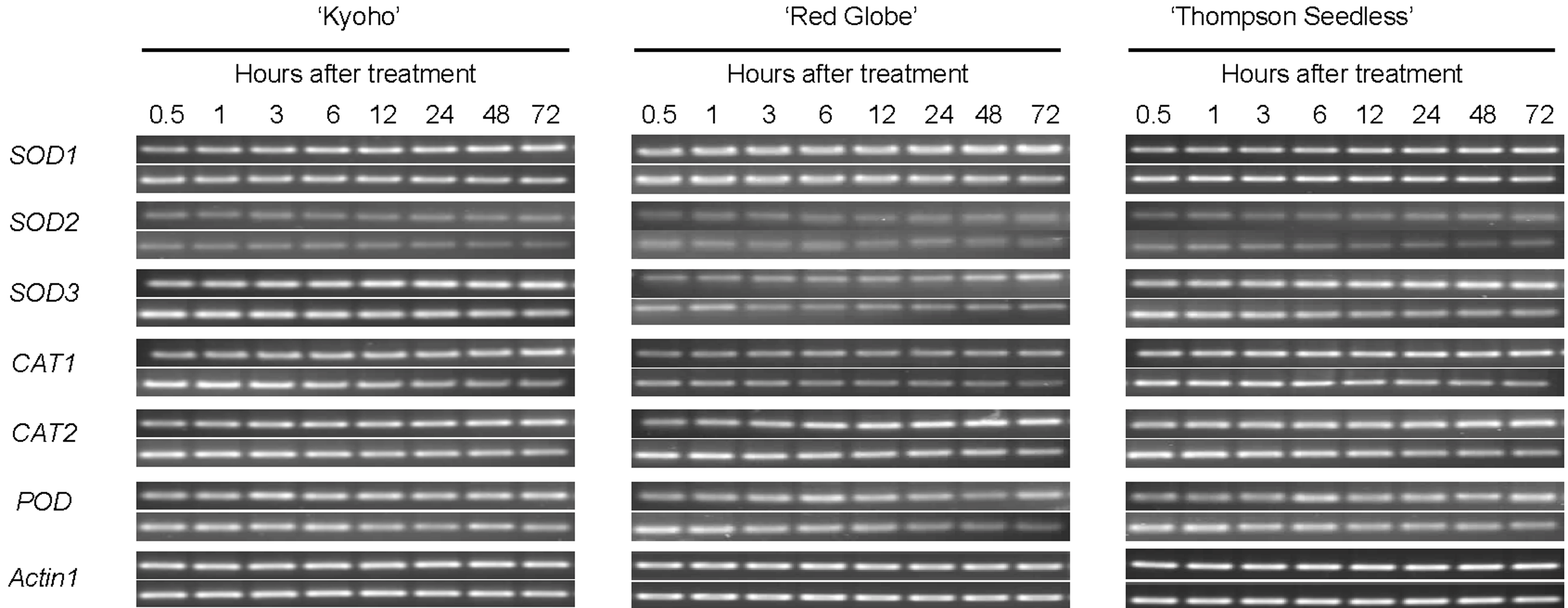

Supplement: Figure S5 — Semi-quantitative RT-PCR analysis of the expression of genes encoding antioxidant enzymes in ‘Kyoho’, ‘Red Globe’ and ‘Thompson Seedless’ flowers following GA3 treatment. For each gene, the bands in the top row represent amplified products from flowers of untreated controls while bands in the bottom row represent amplified products from flowers of GA3-treated samples. (TIFF) [file pone.0080044.s006.tiff]

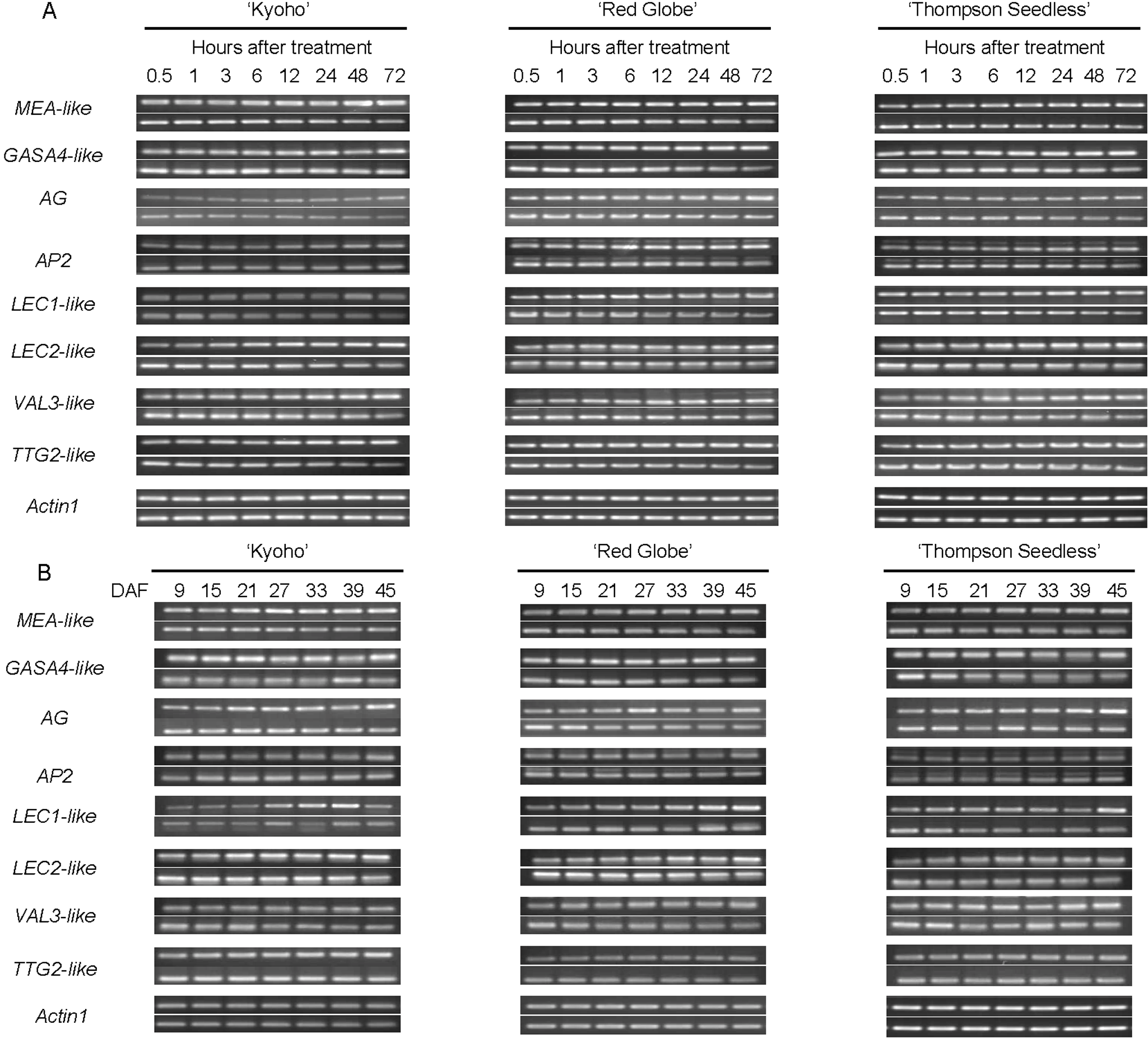

Supplement: Figure S6 — Semi-quantitative RT-PCR analysis of the expression of genes related to seed development in ‘Kyoho’, ‘Red Globe’ and ‘Thompson Seedless’ flowers (A) and seeds (B) following GA3 treatment. For each gene, bands in the top row represent amplified products from flowers and seeds of untreated control samples while bands in the bottom row represent amplified products from flowers and seeds of GA3-treated samples. (TIFF) [file pone.0080044.s007.tiff]
